# Immune-Related Genetic Overlap Between Regional Gray Matter Reductions and Psychiatric Symptoms in Adolescents, and Gene-Set Validation in a Translational Model

**DOI:** 10.3389/fnsys.2021.725413

**Published:** 2021-09-30

**Authors:** Lukas Penninck, El Chérif Ibrahim, Eric Artiges, Victor Gorgievski, Sylvane Desrivières, Severine Farley, Irina Filippi, Carlos E. A. de Macedo, Raoul Belzeaux, Tobias Banaschewski, Arun L. W. Bokde, Erin Burke Quinlan, Herta Flor, Antoine Grigis, Hugh Garavan, Penny Gowland, Andreas Heinz, Rüdiger Brühl, Frauke Nees, Dimitri Papadopoulos Orfanos, Tomáš Paus, Luise Poustka, Juliane H. Fröhner, Michael N. Smolka, Henrik Walter, Robert Whelan, Julien Grenier, Gunter Schumann, Marie-Laure Paillère Martinot, Eleni T. Tzavara, Jean-Luc Martinot, for the IMAGEN Consortium

**Affiliations:** ^1^Institut National de la Santé et de la Recherche Médicale, INSERM U1299 “Trajectoires Développementales en Psychiatrie”, Université Paris-Saclay, Ecole Normale Supérieure Paris-Saclay, CNRS, Centre Borelli, Gif-sur-Yvette, France; ^2^Aix Marseille Univ, CNRS, INT, Inst Neurosci Timone, Marseille, France; ^3^EPS Barthelemy Durand, Etampes, France; ^4^University of Paris, CNRS, INCC, Paris, France; ^5^Centre for Population Neuroscience and Precision Medicine (PONS), Institute of Psychiatry, Psychology & Neuroscience, SGDP Centre, King’s College London, London, United Kingdom; ^6^AP-HM, Hôpital Sainte Marguerite, Pôle de Psychiatrie Universitaire Solaris, Marseille, France; ^7^Department of Child and Adolescent Psychiatry and Psychotherapy, Central Institute of Mental Health, Medical Faculty Mannheim, Heidelberg University, Mannheim, Germany; ^8^Discipline of Psychiatry, School of Medicine and Trinity College Institute of Neuroscience, Trinity College Dublin, Dublin, Ireland; ^9^Institute of Cognitive and Clinical Neuroscience, Central Institute of Mental Health, Medical Faculty Mannheim, Heidelberg University, Mannheim, Germany; ^10^Department of Psychology, School of Social Sciences, University of Mannheim, Mannheim, Germany; ^11^NeuroSpin, CEA, Université Paris-Saclay, Gif-sur-Yvette, France; ^12^Department of Psychiatry and Psychology, University of Vermont, Burlington, VT, United States; ^13^Sir Peter Mansfield Imaging Centre, School of Physics and Astronomy, University of Nottingham, Nottingham, United Kingdom; ^14^Department of Psychiatry and Psychotherapy, CCM, Charité – Universitätsmedizin Berlin, Corporate Member of Freie Universität Berlin, Humboldt-Universität zu Berlin, and Berlin Institute of Health, Berlin, Germany; ^15^Physikalisch-Technische Bundesanstalt (PTB), Braunschweig, Germany; ^16^Institute of Medical Psychology and Medical Sociology, University Medical Center Schleswig Holstein, Kiel University, Kiel, Germany; ^17^Department of Psychiatry, Faculty of Medicine and Centre Hospitalier Universitaire Sainte-Justine, University of Montreal, Montreal, QC, Canada; ^18^Department of Psychology and Psychiatry, University of Toronto, Toronto, ON, Canada; ^19^Department of Child and Adolescent Psychiatry and Psychotherapy, University Medical Centre Göttingen, Göttingen, Germany; ^20^Department of Psychiatry and Neuroimaging Center, Technische Universität Dresden, Dresden, Germany; ^21^School of Psychology and Global Brain Health Institute, Trinity College Dublin, Dublin, Ireland; ^22^Université de Paris, INSERM UMRS 1124, Paris, France; ^23^PONS Research Group, Department of Psychiatry and Psychotherapy, Humboldt University, Berlin and Leibniz Institute for Neurobiology, Magdeburg, Germany; ^24^Institute for Science and Technology of Brain-inspired Intelligence (ISTBI), Fudan University, Shanghai, China; ^25^AP-HP.Sorbonne Université, Department of Child and Adolescent Psychiatry, Pitié-Salpêtrière Hospital, Paris, France; ^26^Fondation Fondamental, Créteil, France

**Keywords:** immunity genes, psychiatric symptoms, adolescence, MRI, childhood maltreatment

## Abstract

Adolescence is a period of vulnerability for the maturation of gray matter (GM) and also for the onset of psychiatric disorders such as major depressive disorder (MDD), bipolar disorder and schizophrenia. Chronic neuroinflammation is considered to play a role in the etiology of these illnesses. However, the involvement of neuroinflammation in the observed link between regional GM volume reductions and psychiatric symptoms is not established yet. Here, we investigated a possible common immune-related genetic link between these two phenomena in european adolescents recruited from the community. Hippocampal and medial prefrontal cortex (mPFC) were defined *a priori* as regions of interest (ROIs). Their GM volumes were extracted in 1,563 14-year-olds from the IMAGEN database. We found a set of 26 SNPs that correlated with the hippocampal volumes and 29 with the mPFC volumes at age 14. We formed two ROI-Related Immune-gene scores (RRI) with the inflammation SNPs that correlated to hippocampal GM volume and to mPFC GM volume. The predictive ability of both RRIs with regards to the presence of psychiatric symptoms at age 18 was investigated by correlating the RRIs with psychometric questionnaires obtained at age 18. The RRIs (but not control scores constructed with random SNPs) correlated with the presence of depressive symptoms, positive psychotic symptoms, and externalizing symptoms in later adolescence. In addition, the effect of childhood maltreatment, one of the major environmental risk factors for depression and other mental disorders, interacted with the RRI effect. We next sought to validate this finding by investigating our set of inflammatory genes in a translational animal model of early life adversity. Mice were subjected to a protocol of maternal separation at an early post-natal age. We evaluated depressive behaviors in separated and non-separated mice at adolescence and their correlations with the concomitant expression of our genes in whole blood samples. We show that in mice, early life adversity affected the expression of our set of genes in peripheral blood, and that levels of expression correlated with symptoms of negative affect in adolescence. Overall, our translational findings in adolescent mice and humans provide a novel validated gene-set of immune-related genes for further research in the early stages of mood disorders.

## Introduction

Some large-scale studies combining genetic and brain structural data hypothesize the existence of shared neurobiological mechanisms underlying prevalent psychiatric disorders, such as major depressive disorder (MDD), attention-deficit/hyperactivity disorder, and schizophrenia (Parker et al., 2020; Patel et al., 2021). The central findings supporting this hypothesis are the associations found between disorder-specific regional differences in brain structure (e.g., cortical thickness or regional volumes) and common clusters of genes involved in brain development or maturation. Although they do not establish causality, these observations point to the interplay of genetic and brain structural underpinnings in the pathophysiology of psychiatric illnesses. Here, we aim to further contribute to this endeavor by applying a targeted approach, i.e., by focusing on a limited number of genes and brain regions. The main advantage of such an approach is the improvement of statistical power to identify associations in smaller samples. Specifically, we will investigate the possible association between neuroinflammatory-related genes and regional gray matter (GM) volumes in the hippocampus and medial prefrontal cortex (mPFC) in the development of mood disorders (MDD and bipolar disorder) and schizophrenia.

Although a wide range of structural abnormalities has been associated with psychiatric disorders (e.g., Patel et al., 2021), herein we focused on volumetric GM measurements in the hippocampus and mPFC as regions of interest (ROI). Indeed, reduced hippocampal and PFC volume are among the most replicated findings in MRI studies of depression (MacQueen et al., 2003; Schmaal et al., 2020). Lower hippocampal volumes have been associated with adolescent onset MDD (Chen et al., 2010; Schmaal et al., 2016), and ventral medial PFC maturation has been related to negative affect in the developing brain (Ducharme et al., 2014). GM reductions in these two regions have been put forward as indicators of the severity and stage of MDD (Belleau et al., 2019). Still, in their review, Belleau et al. (2019) speculate that, although hippocampal and mPFC GM reductions have been associated with MDD, these reductions are neither necessary nor sufficient for inducing a depressive episode. Instead, these structural abnormalities should be regarded as intermediary effectors driving the progression and recurrence of depression. Consistently, our group has reported lower volumes in both regions as variables of interest for tackling irregular sleep habits paving the way to psychiatric symptoms in adolescents (Lapidaire et al., 2021).

Our second reduction in scope, i.e., focusing on a carefully selected set of immune-related genes rather than a genome-wide paradigm, is founded on research putting forward chronic neuroinflammation as a neurobiological characteristic of MDD driving GM loss (Kubera et al., 2011; Barnes et al., 2017). This theory, originally called the “inflammatory and neurodegenerative hypothesis of depression” by Maes et al. (2009), is based on multiple pieces of evidence. The first paper demonstrating a close connection between depression and the immune system was published in 1990 and found that MDD was often associated with a significantly higher number of activated T-cells (Maes et al., 1990). Since then, there have been many consistent findings of increased levels of proinflammatory cytokines in the cerebrospinal fluid (CSF) of patients with depression, the most prominent being interleukin-1 (IL-1), IL-2, IL-6, IL-8, IL-12, interferon-γ and tumor necrosis factor-α. In addition, elevations in peripheral blood concentrations of chemokines, adhesion molecules, acute phase proteins and inflammatory mediators such as prostaglandins have been observed. Lastly, depression could be induced by administrating cytokines (see the comprehensive reviews by Raison et al., 2006 and Miller and Raison, 2015).

To our knowledge, there is no report investigating the putative association between immune-related genetic variation and MDD-related hippocampal and mPFC GM reductions. In order to do so, we will combine the benefits of a candidate gene approach and a polygenic approach. More specifically, we will construct two “polygenic” scores using only inflammation-related common genetic polymorphisms: one in association with hippocampal GM volume and another in association with mPFC GM volume. Hence, these scores will be referred to as ROI-related immune-gene scores (RRI-scores). The IMAGEN database will be used to access genetic and T1 imaging data in 14-year-olds, an age of particular interest as confounding effects due to the use of medication can be considered minimal. A second notable asset of the IMAGEN database is the availability of follow-up data at the age of 18, including a wide range of psychometric data. We will use this follow-up to investigate both RRI-scores with regards to the participants’ psychiatric symptoms later in adolescence. Thus, we will examine whether the putative association between neuroinflammatory genes and regional GM reductions plays a role in the development of psychiatric symptoms.

Moreover, we will test the translational hypothesis that genetic predisposition influences the capacity of an environmental risk factor to induce a psychiatric disorder (Caspi and Moffitt, 2006; Bagot et al., 2014). First, we will assess whether there is an interaction between the RRIs and the degree of childhood maltreatment (CM) explaining negative affects at adolescence, in the IMAGEN database. Second, we will use a translational approach employing an animal model of early life adversity. We will assess whether (i) mice subjected to early life adversity display depressive-like behaviors at adolescence; (ii) the expression of the constructed hippocampal RRI gene-set is altered in peripheral blood in these mice; (iii) transcript levels correlate with the severity of depression-related behavioral scores in adolescent mice. The advantage of this combined approach is that we use transcriptional profiling, which measures the expression of genes and is sensitive to both genotype and environment, to gain insight toward the (patho)physiological link between inflammatory pathways, childhood trauma, and depression symptoms in adolescence.

## Materials and Methods

### Participants

Participants’ datasets were drawn from the IMAGEN project, a European multi-center collaboration combining genetic, neuro-imaging and neuropsychological data from 2223 adolescents at baseline (BL; 14 years old). Participants were followed up 2 years (follow-up 1; FU1) and 4 years later (FU2). An initial sample of 1563 14-year-old adolescents was defined, for which genetic information, T1-weighted MRI images passing the different quality control procedures and the multiple necessary variables were available. In order to perform correlational analyses with psychometric measurements, subgroups of the initial sample were constructed with participants for whom the necessary psychometric data were available. Recruitment procedures have been previously described (Schumann et al., 2010). Written informed consent was obtained from all participants and their legal guardians and verbal assent was obtained from the adolescents.

### Neuro-Imaging Data

#### T1-Weighted MRI

High-resolution T1-weighted anatomical MR images were obtained by means of three Tesla scanners (Philips, Siemens, and General Electric), using a standardized 3D T1-weighted magnetization prepared rapid acquisition gradient echo (MPRAGE) sequence based on the ADNI protocol.^1^ The full details of the MRI acquisition protocols and quality checks have been described previously (Schumann et al., 2010). Image preprocessing was performed with Statistical Parametric Mapping 12 software (SPM12) and its toolbox extension Computational Anatomy Toolbox 12 software (CAT12). In summary, T1-weighted images were segmented and normalized using customized tissue probability maps. Then, the normalized, segmented, and modulated gray matter (GM) and white matter (WM) images were smoothed using a 8-mm full-width at half-maximum Gaussian kernel. Total GM, WM, and cerebrospinal fluid (CSF) volumes were computed for each participant. Total intracranial volume (TIV) was defined as the sum of GM, WM, and CSF volumes. Correct segmentation by CAT12 was verified through visual evaluation of the outliers determined by the automated quality control step “Check Sample Homogeneity” available in CAT12.

#### Extraction of Regional Gray Matter Volumes

The matlab-script “get_totals.m”^2^ was used to extract the hippocampal and mPFC GM volumes from the baseline GM-segmented T1 MRI images. In order to do so, two masks were designed (see Supplementary Figure 1) using WFU Pickatlas software (a SPM12 toolbox extension): a bilateral hippocampal mask, available in Pickatlas, and a mPFC mask, composed of the Brodmann areas (BA) 10, 11, 12, 14, 24, 25, 32, and 33. Here the medial prefrontal cortex was defined in its widest sense. For instance, BA11 was added because it pertains to the orbito-frontal cortex in its most medial part; BA10 pertains to the anterior prefrontal pole but it also includes a medial part.

### Genetic Data

#### SNP Genotyping

The DNA purification and genotyping procedures implemented in the IMAGEN study have been previously described (Desrivières et al., 2015). Population homogeneity was verified with the Structure software using HapMap populations as reference groups (Pritchard et al., 2000). Further correction for population stratification through principal component analysis was deemed unnecessary. After the quality control measures, genotypic data for a total of 466 125 SNPs were considered. The software PLINK was used to extract the SNP genotypes.

#### Construction of RRI-Scores

Based on an extensive literature screening, using the keywords “chronic,” “neuroinflammation,” and “review” in PubMed, genes encoding direct and indirect contributors to neuroinflammation were characterized. To this end, every gene (or protein) that was found to be related to neuroinflammation according to at least three reviews was added to the list. Albeit not systematic, we did consider this procedure to be appropriate for this exploratory study. Including more genes by loosening the constraints might be worth exploring in future research, but will not necessarily lead to RRIs with higher predictive power. The SNPs in and around (±5 kb) the listed immune-related genes were obtained by means of the UCSC Genome Browser^3^ and only those genotyped in the IMAGEN database were selected, a total of 674 SNPs. The methodology used in this study to construct the two RRIs (one associated with hippocampal GM volume, the other with mPFC GM volume) was based on the recent guide put forward by Choi et al. (2020). The effect of every SNP on either the hippocampal or the mPFC GM volume was assessed by performing linear regression analyses in R, using data from the initial sample and the standard *lm* function in R. The dependent variable was the BL GM volume of either the hippocampus or the mPFC, the independent variable was the major allele count for the SNP of interest (0 = homozygous for the minor allele, 1 = heterozygous, 2 = homozygous for the major allele). The regression was controlled for the covariates sex, Puberty Developmental Scale (PDS) score as a proxy of age, TIV and scanner type. Next, the SNPs that correlated (*p* < 0.1 without correction for multiple comparisons) with the hippocampal or mPFC GM volume were selected. In order to control for linkage disequilibrium (LD) and avoid redundancy in the SNPs included in the score, a manual procedure analogous to SNP pruning was carried out. More specifically, the online application LDmatrix developed by the National Institute of Health^4^ was used to study the LD between the SNPs. Groups of SNPs that were found in high LD (*r*^2^ > 0.5) were replaced by the most significantly correlated SNP of that group as representative, eliminating the other SNP(s) of that group. Lastly, for every participant, a hippocampal RRI (HRRI) and mPFC RRI (MRRI) were calculated based on the participant’s genotype for the group of independent SNPs that were correlated with the hippocampal and mPFC GM volume, respectively. More precisely, the score was defined by the sum of minor alleles for the included SNPs, weighted by the effect size of those alleles individually. A normalization of the HRRI and MRRI values was performed in order to obtain two scores ranging from 0 to 10 that could easily be compared and combined.

#### Construction of Control Scores

A collection of 674 SNPs available in the IMAGEN database was randomly selected. Using an identical methodology as described above, a control score explaining hippocampal GM volume and a control score explaining mPFC GM volume were designed using these 674 random SNPs.

### Questionnaire Data

#### Questionnaires

Five questionnaire measurements were extracted from the IMAGEN database. First, the algorithmically calculated scores (ranging from 0 to 5) representing the probability of depression according to the DSM-IV (referred to as DepBand) were obtained for participants at FU2 through the Development and Well-Being Assessment (DAWBA), a self-administered diagnostic questionnaire.^5^ Second, the Community Assessment Psychic Experiences-42 (CAPE-42) questionnaire was used to obtain three scores evaluating the presence of depressive symptoms (referred to as the Depressive Dimension Score; DDS) as well as psychotic experiences, both positive (Positive Dimension Score; PDS) and negative (Negative Dimension Score; NDS) at FU2. Third, the self-reported Strengths and Difficulties Questionnaire (SDQ) at FU2 was used to construct two scores representing externalizing and internalizing behaviors; the Externalizing Score (ES) by summing the Conduct Problems Score and Hyperactivity Score, the Internalizing Score (IS) by summing the Emotional Symptoms Score and the Peer Problems Score. Fourth, a score representing childhood maltreatment (CM) was constructed based on information from the Childhood Trauma Questionnaire (CTQ). This retrospective recall-based questionnaire was administered to the participants at BL and, as described in the manual, produces a score (ranging from 0 to 4) representing the endured stress with regards to six categories. Participants were subsequently categorized in five groups based on the highest score in the six subscales. Ultimately, the three highest groups were merged, resulting in a CM score ranging from 0 to 2. Fifth, the Alcohol Use Disorders Identification Test (AUDIT) Score at FU2 was extracted from the database in order to be included as a covariate in linear regression analyses modeling FU2 GM volumes.

#### Correlational Analyses

The above-mentioned psychometric measurements (DepBand, DDS, PDS, NDS, ES, and IS) were modeled separately in function of the HRRI, the MRRI and the sum of both scores (HMRRI), as well as the control scores. These regression analyses were performed in R. Since the dependent variables DepBand, ES and IS displayed probability distributions similar to a Poisson distribution, Poisson regression (the log-linear type of the generalized linear model) was opted for. The dependent variables DDS, PDS, and NDS were found normally distributed. However, a log-transformation of the dependent variable was implemented in order to correct for the positive skewness. All regression analyses were controlled for the covariates gender and CM. Also, the interaction between CM and the score was evaluated. *P*-values were corrected for multiple comparisons through the Benjamini-Hochberg false discovery rate (FDR) procedure, using the *p.adjust* function in R.

### Animals

All experiments on mice were carried out according to policies on the care and use of laboratory animals of European Community legislation 2010/63/EU. The local Ethics Committee (Comité d’éthique en expérimentation animale Charles Darwin N°5) approved the protocols used in this study (protocol number 01486).

The mice were kept under standard conditions at 22 ± 1°C, and a 12-h light-dark cycle with food and water available *ad libitum.*

### Maternal Separation/Maternal Stress Protocol

Pregnant dams (BALB/c Jico) were purchased from Centre d’Elevage Janvier, (Le Genest St Isle, France) to arrive in our facility 5 days before expected delivery. Dams and their respective litters were divided into two groups. The first group (MS, *Maternaly separated; n = 3 dams*) was subjected to maternal separation/maternal stress procedure; the second group (NS, *No Separation; n = 2 dams*) of dams and respective litters was kept in standard housing conditions as controls.

The maternal separation/maternal stress protocol was adapted from Franklin et al. (2010). The protocol combined (i) physical separation of the pups from the mother and among them; (ii) a short maternal stress at the end of the separation period; (iii) unpredictability regarding the timing of the separation and the maternal stressors.

For maternal separation the pups were placed in separate clean compartments inside a temperature- and humidity-controlled terrarium, to avoid any physical distress of the pups; the mother was placed in a clean novel cage with bedding, food, and water. Maternal separation lasted for 3 h and was applied once daily from post-natal day 1 (P1) to P14; the timing was unpredictable. Maternal stress was applied to the dam at the end of the 3 h separation period and consisted of one of the following: 20 min contention in a plastic perforated tube; 10 min forced swimming stress; 10 min tail-suspension stress. Stressors were alternated pseudorandomly. Both MS and NS groups were left undisturbed between P14 and P21 (weaning). At weaning the sex of the pups was determined (for the present cohort: 6 male and 17 female) and they were subsequently assigned to social groups of 3–4 mice per cage, composed of animals of same sex, and subjected to the same protocol (MS or NS), but from more than one dams to avoid litter effect. The sex ratio per group was, for MS: 4M/9F; and for NS: 3M/7F. A Fisher’s exact test applied to these sex ratios is not statistically significant (*p* > 0.999).

### Behavioral Characterization of the Pups at Late Adolescence

We evaluated behaviors associated with depression (anhedonia, anxiety) in the separated (*n* = 13) and non-separated (*n* = 10) pups at late adolescence (P52–59). Behavioral dimensions were assessed with the Sucrose preference (anhedonia; P52) and Dark-light tests (anxiety; P59).

#### Sucrose Preference

For the sucrose preference test mice were first habituated to drink from two graduated pipettes one filled with water, and the other with sucrose solution for 3 days, the side of the sucrose pipette being alternated each day. On day 4 and after an overnight (15 h) deprivation of water, the two pipettes were presented again; one was filled with water and the other with 4% sucrose. The water and sucrose solution consumed over a 3 h-period, were measured. The sucrose preference index is defined as (sucrose consumed)/(sucrose consumed + water consumed) × 100 (percentage index).

#### Dark-Light

The apparatus consisted of one box divided in two compartments, an illuminated one (30 × 20 × 20 cm), which is open, and a dark one (15 × 20 × 20 cm), which is covered with a lid. A small aperture (width of 5.5 cm and height of 7 cm) allows the mouse to freely move between the dark compartment and the illuminated one. At the beginning of the experiment, the mouse was placed in the illuminated box, facing the aperture. Time spent in the lit box was measured during a 10 min period.

Next, we performed a *Z*-normalization. For this an individual *z*-score was calculated for each test and for each animal as follows: *z*-score = [(individual data for observed parameter) - (mean of control group)]/(standard deviation of control group). For both sucrose preference and dark/light *z*-scores were multiplied by −1, as decreased sucrose preference and decreased time in lit compartment measure depressive/anxiety-like behaviors. For the computation of means and standard deviation of control groups, control groups were defined as NS mice; note that for control groups the mean of *z*-score by behavioral dimension is equal to zero. Subsequently, a global “depression-index” for each animal was calculated by averaging the *z*-scores of the two individual tests as previously described (Apazoglou et al., 2018).

### Blood Collection

At the end of the behavioral evaluations (P60), 0.25 ml of blood was collected from the submandibular vein and stabilized with 1.3 ml RNAlater^®^ solution (Life Technologies, Ambion, Austin, TX). Mice were euthanized several months later by pentobarbital injection.

### RNA Isolation

Total RNAs were purified from the blood using the Mouse RiboPure-Blood RNA isolation kit (Invitrogen), according to manufacturer’s recommendations. After washings, total RNAs were eluted with 0.1 mM EDTA and were subsequently submitted to DNase treatment (DNA-free^TM^ kit, Life Technologies, Ambion, Austin, TX). RNA concentration was determined using a nanodrop ND-1000 spectrophotometer (Thermo Scientific, Waltham, MA). Three samples (one female MS/two male NS) did not provide enough quantities and qualities and were further excluded from the gene expression analysis.

### Candidate mRNA Expression Quantification

850 ng of total RNA was reverse transcribed using the High-Capacity cDNA Reverse Transcription kit (Life Technologies, Applied Biosystems, Foster City, CA). 800 ng of the resulting cDNA were combined with a TaqMan^®^ Fast advanced Master Mix (Thermo Fisher Scientific) and real-time PCR reactions were simultaneously run in triplicate in a thermocycler under the following conditions: 2 min at 50°C, 10 min at 92°C, 45 cycles of 1 s at 95°C, and 20 s at 60°C, in custom array microfluidic cards (Applied Biosystems, Pleasanton, CA) using a QuantStudio^TM^ 7 system and data collected using QuantStudio^TM^ Real-Time PCR Software v1.1 (Applied Biosystems). For each of the 16 candidate genes tested (related to the SNPs included in the HGPS), primer-probe sets were selected using the web portal of the manufacturer (Applied Biosystems, see Supplementary Table 5). In addition, *Rab5a* was universally used as a reference gene (Hervé et al., 2017). Raw *Ct* values were obtained with manual baseline settings on the ThermoFisher cloud RQ software (Applied Biosystems), and then the relative expression level of each mRNA was quantified by using the 2^–ΔΔ*Ct*^ method (Livak and Schmittgen, 2001). In this method, each candidate gene is quantified relative to the expression of *Rab5a* and each amplification is also compared to a calibrator sample (the mean of the samples from the NS mice).

### Statistics

#### Behavioral Evaluation

The *z*-scores for anhedonia, dark light, and the global depression-like index were analyzed separately. For each parameter, the comparison of two independent groups (MS vs. NS) involved Student’s *t*-test that were performed using the STATISTICA software. The results are expressed as mean ± SEM (standard error).

#### Gene Expression

For gene expression comparisons, after observing non-homogeneous variances for each candidate gene in each subgroup (through the [R] function *levene_test*) and absence of normality of residuals (through the [R] function *shapiro_test*) of a parametric model (through the [R] function *lm*), a non-parametric (by permutation) equivalent of a two-way factorial ANOVA was performed through the [R] function *aovp* in the *lmPerm* library. When ANOVA effects were significant, multiple group comparisons for each gene were performed through the [R] function *pairwise.perm.t.test* in the *RVAideMemoire* library to provide FDR *p*-value adjustment.

#### Behavior and Gene Expression Correlations

Correlograms, allowing visualization of behavior and gene expression data into correlation matrices were implemented through the [R] function *corrplot* in the *corrplot* library and variables were ordered according to first principal components. Linear regressions with 95% confidence intervals were plotted through the [R] functions *ggplot, geom_point, and geom_smooth* (with “lm” method) in the *ggplot2* library.

## Results

### Selection of Genes and SNPs

Supplementary Table 1 lists the 90 immune-related genes selected for this study and classifies them in 6 categories: “Cytokines and Cytokine Receptors” (43 genes), “Oxidative Stress Effectors” (4), “Monocytosis and Granulopoiesis” (14), “Inflammatory Signaling Pathway” (21), “Kynurenine Pathway” (5), and “Phospholipases” (3). For these 90 genes, 674 related common SNPs were found genotyped in the IMAGEN database.

### The Single Effects of Immune-Related SNPs on Brain Structure

In our initial sample of 1563 14-year-old participants, the mean bilateral hippocampal GM volume was 1.20 ± 0.109 ml and the mean mPFC GM volume was 30.1 ± 3.42 ml. Other volumetric data from the 14-year-olds (BL) and 18-year-olds (FU2) can be found in Supplementary Table 2. Sex, PDS, TIV and scanner type were all found to be significantly reacted with GM volume (*p* < 0.001), regardless of the region. The individual effects of the selected 674 SNPs on BL hippocampal and mPFC GM volume were assessed through linear regression analyses, controlled for sex, PDS, TIV, and scanner type. No correlation surpassed the significance threshold of *p* < 0.05 after Bonferroni correction for multiple comparisons.

### Construction of the RRIs

As described in the section “Materials and Methods,” we constructed two scores, one explaining the hippocampal GM volume at BL (HRRI), the other explaining the mPFC GM volume at BL (MRRI), using only immune-related SNPs. We found 26 “independent” SNPs that were considerably correlated with the hippocampal volume and thus incorporated in the HRRI; 29 SNPs were combined in the MRRI (Supplementary Tables 3, 4).

### Correlation of the RRIs With Psychometric Data

**FIGURE 1:**
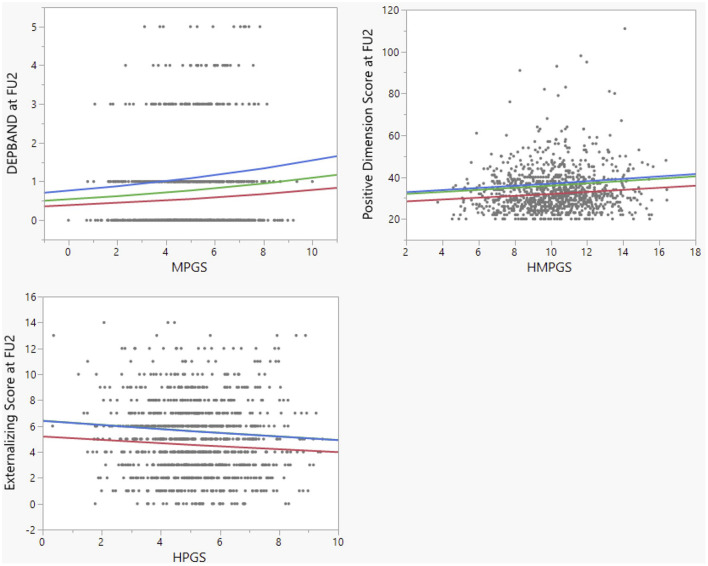
Correlations between RRIs (determined from their link with GM regional volumes at age 14 as described in the text) and psychometric measurements at age 18. MPGS = MRRI, HPGS = HRRI, HMPGS = HMRRI. The colored lines are proposed regression lines for the different levels of childhood maltreatment (CM), after controlling for CM and sex. Red: CM = 0; green: CM = 1; blue: CM = 2. The blue and green line overlap in the bottom left graph.

The first psychometric measurement of interest was the DepBand, representing depression probability at FU2 (Table 1). Controlling for the covariates sex and CM, DepBand, could not be correlated with the HRRI [*p(corr)* = 0.721]. The MRRI, however, positively and significantly covaried with DepBand [*p(corr)* = 0.0296; Figure 1]. Since both scores were not found redundant (*p* = 0.436), we also created the HMRRI by adding up the HRRI and MRRI. The HMRRI positively covaried with DepBand [*p(corr)* = 0.0536] but did not survive FDR-correction. Next, the interactions between CM and the scores in relation to DepBand were evaluated. The number of participants with a CM score of 0 was 588, 283 had a score of 1, and 108 had a score of 2. A positive and significant interaction was observed between the HRRI and childhood maltreatment score CM [*p(corr)* = 0.00327]. This suggests that, as the level of endured trauma increases, the association between MRRI and presence of depressive symptoms at age 18 increases as well. It is important to note that all goodness-of-fit Chi-Squared tests for the Poisson regressions were found significant, suggesting that the data do not fit the model perfectly well. Alternative models were considered but not found better.

**TABLE 1:** Correlations between depression probability (DAWBA score) at age 18 and the genetic scores.

	Independent variable	*χ^2^* (df)	*B*	*z*	*p*	*p (corr)*
**DepBand at FU2**	HRRI	1192 (857)	0.0207	0.770	0.441	0.721
	MRRI	1185 (857)	0.0764	2.811	0.00494	0.0296*
	HMRRI	1186 (857)	0.0477	2.518	0.0119	0.0536
	CM:HRRI	1180 (856)	0.122	3.57	0.000364	0.00327*
	CM:MRRI	1184 (856)	0.0271	0.735	0.463	0.556
	CM:HMRRI	1179 (856)	0.0679	2.80	0.00516	0.0310*

*The DepBand score (DAWBA questionnaire) obtained at FU2 was correlated with the three RRIs (ROI-Related-Immune-gene-score) as well as the three interactions between the scores and childhood maltreatment (CM) by means of a Poisson regression (*N* = 861).*

**χ2* (*df*) = residual deviance (degrees of freedom), used for Chi-Squared goodness-of-fit test; *B* = unstandardized regression coefficient.*

* *p* < 0.05 (FDR-corrected).

The second psychometric tool of interest was the CAPE-42, which consists out of three subscores (Table 2). A positive and significant correlation was observed between the log-transformed Positive Dimension Score, representing the amount of positive psychotic symptoms at FU2, and the HMRRI [*p(corr)* = 0.0296; Figure 1]. As no significant interactions were found between CM and the RRIs in explaining one of the three CAPE-42 subscores, these results are not shown in Table 2.

**TABLE 2:** Correlations between the clinical dimensions (CAPE-42 subscores) and the genetic scores.

	Independent variable	*R* ^2^	*B*	*t*	*p*	*p (corr)*
Positive Dimension (log)	HRRI	0.0449	0.0115	2.01	0.0444	0.114
	MRRI	0.0466	0.0134	2.38	0.0175	0.0630
	HMRRI	0.0506	0.0124	3.11	0.00192	0.0296*
Negative Dimension (log)	HRRI	0.0431	–0.00887	–1.06	0.289	0.520
	MRRI	0.0420	0.0087	0.226	0.821	0.921
	HMRRI	0.0423	–0.00342	–0.583	0.560	0.840
Depressive Dimension (log)	HRRI	0.091	0.000741	0.091	0.928	0.928
	MRRI	0.0933	0.0124	1.54	0.123	0.277
	HMRRI	0.0952	0.00667	1.161	0.246	0.492

*The three subscores of the CAPE-42 questionnaire obtained at FU2 were correlated with the three RRIs after log-transformation of the dependent variables (*N* = 930).*

**R*^2^ = coefficient of determination; *B* = unstandardized regression coefficient.*

* *p* < 0.05 (FDR-corrected).

Lastly, the Externalizing Score (ES) and Internalizing Score (IS) at FU2 were modeled in function of the scores, again using Poisson regression (Table 3). A significant inverse correlation was observed between the ES and the HRRI [*p(corr)* = 0.0294; Figure 1]. We also found a significant positive interaction between HRRI and CM in explaining the ES [*p(corr)* = 0.000857].

**TABLE 3:** Correlations between Externalizing and Internalizing Score (SDQ) at age 18 and the genetic scores.

	Independent variable	*χ^2^* (df)	*B*	*z*	*p*	*p (corr)*
Externalizing at FU2	HRRI	1563 (951)	–0.0275	–2.90	0.00368	0.0294*
	MRRI	1572 (951)	–0.00262	–0.282	0.778	0.921
	HMRRI	1567 (951)	–0.0146	–2.22	0.0263	0.079
	CM:HRRI	1547 (950)	0.0538	4.07	4.76 × 10^–5^	0.000857*
	CM:MRRI	1572 (950)	–0.00832	–0.610	0.542	0.588
	CM:HMRRI	1561 (950)	0.0228	2.44	0.01473	0.0529
Internalizing at FU2	HRRI	1824 (951)	–0.00465	–0.471	0.637	0.844
	MRRI	1825 (951)	–0.00158	–0.164	0.870	0.921
	HMRRI	1824 (951)	–0.00305	–0.445	0.657	0.845
	CM:HRRI	1819 (950)	0.0298	2.21	0.0269	0.0807
	CM:MRRI	1824 (950)	–0.00118	–0.0850	0.932	0.932
	CM:HMRRI	1822 (950)	0.0143	1.51	0.132	0.237

*The Externalizing and Internalizing Score (SDQ) obtained at FU2 were correlated with the three RRIs as well as the three interactions between the RRIs and childhood maltreatment (CM) by means of a Poisson regression (*N* = 955).*

**χ2* (*df*) = residual deviance (degrees of freedom), used for Chi-Squared goodness-of-fit test; *B* = unstandardized regression coefficient.*

* *p* < 0.05 (FDR-corrected).

### Correlation of the Control Scores With Psychometric Data

No significant correlations were found between the control scores and the different psychiatric symptom measurements. In addition, we did not observe any significant interactions between childhood maltreatment and the control scores in function of the psychiatric symptoms.

### Behavioral Effects of Early Life Stress in Adolescent Animals

**FIGURE 2:**
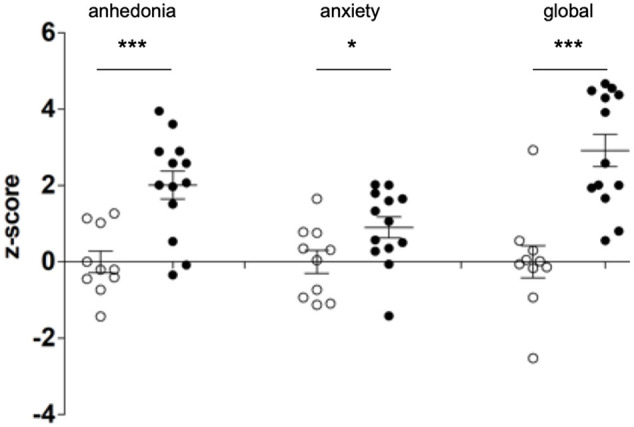
Early life stress induces depressive-like behaviors in adolescent mice. Newborn mice were either subjected to a maternal separation paradigm between P1 (post-natal day 1) and P14 (MS, black dots) or were left undisturbed (controls, NS, white dots). NS and MS mice were evaluated for anhedonia and anxiety (measured in the sucrose preference and the dark-light tests, respectively) in late adolescence, between P52 and P59. Anhedonia and anxiety scores were *z*-transformed and a composite depression-index (global *z*-score) was averaged. Two-tailed Student’s *t*-test shows increased levels of anhedonia (*df* = 21; *t* = 4.155) and anxiety (*df* = 21; *t* = 2.202) as well as increased depression index (*df* = 21, *t* = 4.797) in MS mice as compared to NS. * *p* < 0.05; ***p < 0.0001.

Supplementary Figures 2, 3 as well as Figure 2 show that early life stress profoundly affects anhedonia and anxiety at late adolescence in mice. At late adolescence, mice subjected to early life stress between P1 and P14 show increased anhedonia and anxiety as compared to NS mice (raw data are presented in Supplementary Figures 2, 3 and *z*-score in Figure 2). The global depression-index (Figure 2) is also significantly elevated in MS mice indicating that early life adversity (P1–P14) induces long-lasting negative affects still present in late adolescence (P52–P59).

### Effect of Early Life Stress on Candidate Gene Expression in Adolescent Animals

**FIGURE 3:**
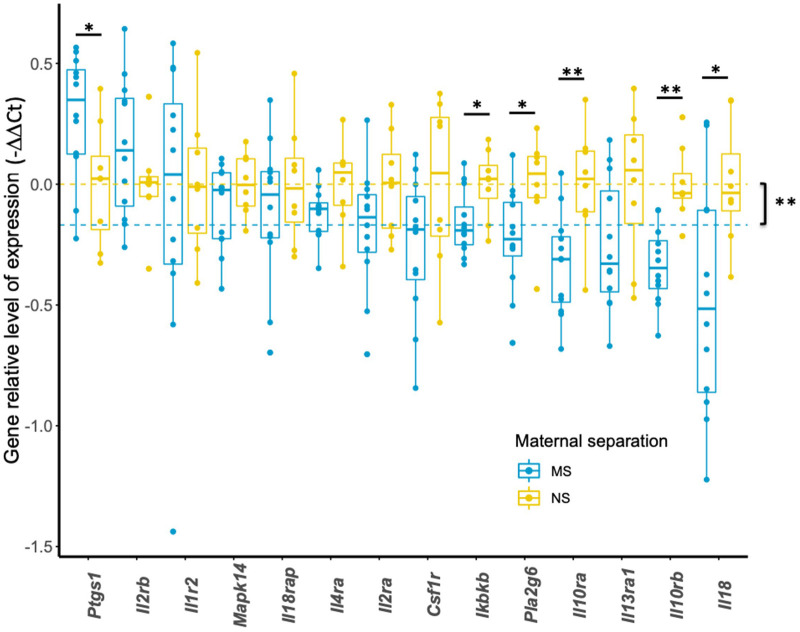
Transcriptional expression of HRRI candidate genes in adolescent mice. Total RNA from blood of NS and MS mice at P60 was profiled by RT-qPCR for 14 HRRI candidate gene transcripts. The expression of each transcript was quantified relative to the expression of a reference gene, *Rab5a*, whereas the mean of NS mice was used as a calibrator. Statistical analysis was realized using a permutation-based non-parametric factorial ANOVA. Yellow and blue dashed lines indicate the mean values of all NS and MS, respectively, and highlight a significant “gene” effect between NS and MS animals (*p* = 0.00295). Significant multigroup comparisons for each gene were performed by pairwise permutation *t*-tests and are as follows, *Ptgs1* (*p* = 0.022), *Ikbkb* (*p* = 0.028), *Pla2g6* (*p* = 0.022), *Il10ra* (*p* = 0.008), *Il10rb* (*p* = 0.002), *Il18* (*p* = 0.032). * *p* < 0.05; **p < 0.01.

We next sought to investigate the effect of early life stress on the transcriptional expression of HRRI set of genes. Among the 17 candidate genes, 3 were not analyzed further (*IKBKG*, *IL12B*, *IL13*) because of low quality results. The remaining 14 candidate genes were well expressed in mouse blood and subsequently constituted the focus of our analysis as “mouse HRRI.” Figure 3 shows that maternal separation has a significant effect on the global transcript level of mouse HRRI (*p* < 2.0E-16). Individually, *post-hoc* analysis demonstrated significant dysregulation for *Ikbkb*, *Il10ra*, *Il10rb*, *Il18*, *Pla2g6*, and *Ptgs1*. Among those *Ptgs1* was increased while all the others were decreased in MS mice.

### Correlations Between Gene Expression and Behavior in Adolescent Mice

**FIGURE 4:**
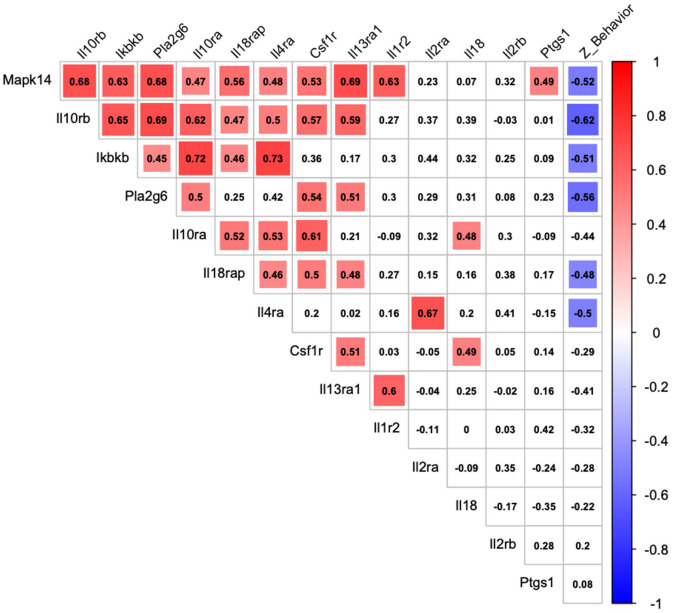
Correlation between transcriptional expression of HRRI candidate genes and depressive-like behavior in adolescent mice. Correlation matrix graph, correlogram, highlighting correlation between qualitative (*Z*_Behavior, a depression-index reflecting a composite score of anhedonia and anxiety subscores) and quantitative (transcriptional relative level of HRRI candidate genes obtained by RT-qPCR from blood samples) variables in adolescent mice. The variables are ordered according to first principal components. Positive correlations are displayed in red and negative correlations in blue color. The intensity of the color and the size of the squares are proportional to the Pearson correlation coefficients. Only significant correlations are indicated by a colored square.

**FIGURE 5:**
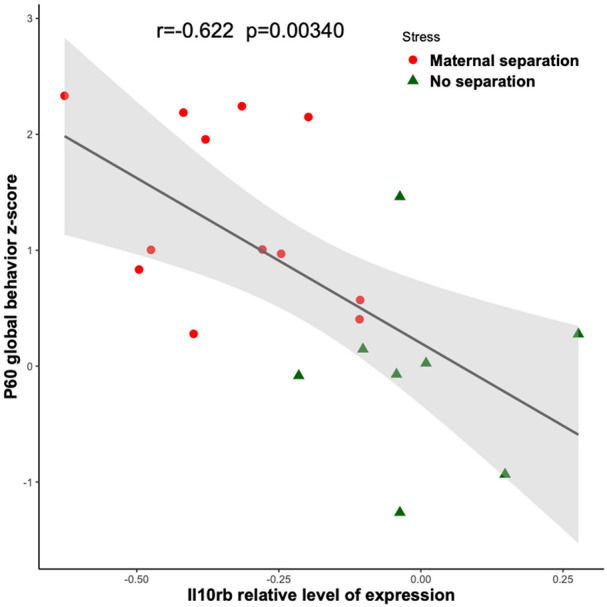
Link between *Il10rb* relative level of transcriptional expression and depressive-like behavior. Linear regressions with 95% confidence intervals (in gray) are plotted between the depression-index and the *Il10rb* transcriptional relative level obtained by RT-qPCR from blood in MS (red circles) and NS (green triangles) adolescent mice. The Pearson correlation coefficient and the associated *p*-value are indicated.

Figure 4 shows that among the 14 mouse HRRI genes profiled, 6 were correlated with the global depression-index (a composite score of anhedonia and anxiety subscores). We noted that all the genes that correlated with behavior, also highly correlated with each other. Figure 5 illustrates the tight link between one of the most significant altered genes, *Il10rb*, and depressive-like behavior. Regression analyses for behavior and *Ikbkb*, *Il10rb*, *Il18*, *Pla2g6*, and *Ptgs1* are shown in Supplementary Figure 4.

## Discussion

In this translational study, we explored the link between inflammation-related genes and brain structure, along with early life adversity and emergence of psychiatric symptoms. We investigated imaging genetics in a large database of community adolescents. We formed scores aggregating inflammation genes related with hippocampal or mPFC volumes at age 14. We found that these RRIs related with psychiatric symptoms at age 18. Also, we found a set of inflammation genes that were related to gray matter volume of hippocampal regions, and to childhood maltreatment score in these adolescents. Expression levels of inflammation genes associated with psychiatric symptoms (genes from HRRI) were subsequently examined in a murine model of early life adversity. Interestingly, expression of these genes was significantly altered after maternal separation in mice.

Excessive activation of the immune system as well as abnormalities in brain structure have been associated with depression. Furthermore, depression as well as its associated structural abnormalities are relatively heritable. Meta-analyses have found a heritability of 37% for depression (Sullivan et al., 2000) and a heritability between 40 and 70% for hippocampal volume (Gu and Kanai, 2014). Yet, characterization of the contributing individual genetic factors has proven to be very difficult. In a large genome wide association (GWA) study that investigated how common genetic variants affect the structure of subcortical regions, only a couple of genetic loci could be significantly correlated (Hibar et al., 2015). Similarly, despite considerable success within other illnesses such as diabetes and rheumatoid arthritis, GWA analyses of MDD have overall failed to produce results at the SNP level (Bogdan et al., 2017). Explanations for this could be found in the phenotypic heterogeneity, the lack of very large sample sizes and the complex functional architecture of the genetic polymorphisms. This last issue has been addressed by exploring gene-environment interactions and applying polygenic approaches. For example, in a recent study, structural abnormalities in schizophrenia were explored by creating a polygenic risk score (PRS) based on the weighted effects of SNPs found associated with schizophrenia in a prior GWA study (Alnæs et al., 2019). Also, PRSs for bipolar disorder and PRSs for schizophrenia both were found to have certain predictive power with regards to depression, corroborating prior evidence that these disorders share some common genetic overlap (Musliner et al., 2019).

Since psychiatric disorders as well as their associated structural abnormalities seem to involve a genetic contribution, we hypothesized the existence of an immune-related genetic overlap between GM structural reductions and psychiatric symptoms. This was explored by constructing two RRIs based solely on SNPs related to inflammatory genes: one predicting hippocampal GM volume in 14-year-olds (HRRI), the other predicting mPFC GM volume (MRRI). We found that both scores were correlated with the presence of different symptoms later in adolescence.

We observed that the RRIs describing the genetic variation in less than 30 inflammatory SNPs had small yet significant predictive power regarding certain psychometric measurements obtained at age 18. As expected, effect sizes were consistently relatively small. The MRRI was found correlated with the presence of depressive symptoms. Secondly, both HRRI and MRRI were correlated with positive psychotic symptoms. Schizophrenia, the main disorder linked with these symptoms, has been consistently associated with neuroanatomical abnormalities such as reductions in GM volume (Weinberger, 1987; Keshavan et al., 2005; Bakhshi and Chance, 2015). Thirdly, we found a negative relationship between the HRRI and externalizing symptoms at age 18. This seems unexpected at first sight. A recent review addressing neuro-imaging findings in two of the major externalizing disorders, conduct disorder and oppositional defiant disorder, did not describe any studies reporting increases of hippocampal GM volume (Noordermeer et al., 2016). However, functional deficiencies in the amygdala, common in externalizing disorders, could be explained by abnormalities in the neighboring hippocampal complex (Yang and Wang, 2017). Lastly, we observed positive interactions between the scores and childhood maltreatment. This means that the probability of developing certain psychiatric symptoms due to a history of childhood maltreatment will be larger in the context of a specific genetic predisposition, in this case a high RRI. Gene-environment interactions in psychiatric diseases have been described repeatedly. For example, a polymorphism in the promoter region of the serotonin transporter gene was reported to moderate the influence of stressful life events on depression (Caspi et al., 2003).

The ability of the RRIs to predict to a limited extent the presence of psychiatric symptoms suggests the existence of the proposed genetic overlap. Indeed, the same variation in immune-related genes was found to explain both GM volume reductions in the hippocampus or mPFC and the degree of certain psychiatric symptoms. However, it could be argued that the RRIs predictive ability is solely due to the fact that the RRIs are constructed in such a way that they represent a portion of the GM volume variance. As the link between GM volumes and psychiatric illnesses is already established, the ability of an alternative score representing those structural abnormalities to predict psychiatric symptoms would not be surprising. In order to investigate this, we performed a control study by constructing two scores on the basis of random SNPs. These control scores did not significantly correlate with any psychometric measurement, nor did they display any interactions with childhood maltreatment. This higher predictive power of the non-random RRIs points at the involvement of the immune system. We thus not only corroborate prior evidence for the link between structural GM reductions and psychiatric illnesses, but also provide pioneering evidence strongly suggesting an immune-related genetic overlap between regional GM volumes and psychiatric symptoms, and define a novel combination of genes involved in this link. In order to further investigate this suggested causality, it would be interesting to perform a longitudinal study in which brain structural changes during adolescence are associated with the development of psychiatric symptoms.

To further investigate the functional importance of the novel gene-set that we defined we employed a translational approach linking genotype and gene expression analyses. Indeed, the phenotype during adolescence is likely to be modulated by both genotype and environment, so that genotype analyses alone probably cannot account for their interaction (Kent et al., 2012). In contrast, transcriptional profiling that measures the expression of genes is sensitive to both genotype and environment and therefore may offer insights in pathophysiology. We focused on blood transcriptomics, since blood signature demonstrated that it could represent a surrogate for brain gene expression and may predict stress-induced behaviors (Sullivan et al., 2006; van Heerden et al., 2009; Rollins et al., 2010; Tylee et al., 2013; Witt et al., 2013; Luykx et al., 2016; Hervé et al., 2017).

We implemented an animal model of early life adversity and measured depression-like behaviors in adolescence. Based on the above reported effects in human subjects we hypothesized that early life adversity would affect not only behavior but also the expression of our set of genes, and that expression would correlate with symptoms of negative affects at adolescence.

Mice were subjected to a protocol of early life adversity (maternal separation) at an early post-natal age (P1–P15). We evaluated behaviors associated with depression (anhedonia: sucrose preference; anxiety: dark-light box) in MS and NS mice at adolescence (P52–P59). We measured the expression of our genes in whole blood samples collected at the same time-point (P60). We specifically focused on the hippocampal gene-set since the hippocampus is a region consistently implicated in depression and depression-like phenotypes in humans and mice (Vythilingam et al., 2002; Turecki et al., 2012; Apazoglou et al., 2018).

Our results showed that mice subjected to early life adversity displayed negative affects at adolescence (Figure 2). The expression of our mouse HRRI gene-set was altered in mice subjected to early life adversity (Figure 3), and transcript levels inside this gene-set correlated with depression-related behavioral score at adolescence (Figure 4).

Within the examined gene-set, Figure 3 shows a significant decrease for *Ikbkb, Il10ra, Il10rb, Il18, Pla2g6*, and an increase for *Ptgs1* transcripts.

Figure 4 shows that among these genes, three (*Ikbkb, Il10rb, Pla2g6)* were significantly correlated with the global depression-index in MS mice. Notably the three also highly correlated with each other.

Interestingly, *Ikbkb, Il10rb, and Pla2g6*, which were decreased in MS mice are implicated in inflammatory homeostasis. *Ikbkb* is a regulator of the canonical NF-Kappa-B pathway a key-pathway in immunity/inflammation (Schmid and Birbach, 2008); *Il10rb* encodes for the anti-inflammatory cytokine IL10 (Shouval et al., 2014), and mice invalidated for IL10 show increased depressive-like behaviors (Mesquita et al., 2008); *Pla2g6* encodes for the iPLA2β protein, which regulates an overall anti-inflammatory response and whose dysregulation is associated with neurogenerative disorders (Guo et al., 2018).

The present findings suggest an inflammatory network of genes that most likely is involved in “depression-associated” neuroinflammatory adaptations in the periphery and CNS. We propose that early stressors like adversity can trigger an imbalance between anti-inflammatory and pro-inflammatory transcripts that may be at the origin of psychiatric symptoms in adolescence. These transcripts might provide both clinical biomarkers and novel targets in understanding and preventing individual developmental trajectories of psychiatric vulnerability.

## Data Availability Statement

The data analyzed in this study is subject to the following licenses/restrictions: The datasets used for analysis can be acceded through requests to the last two authors. Requests to access these datasets should be directed to J-LM and ET, Jean-luc.martinot@inserm.fr and eleni.tzavara@inserm.fr.

## Ethics Statement

The studies involving human participants were reviewed and approved by the Ethics committee CPP IDF 7, le Kremlin Bicètre, France. Written informed consent to participate in this study was provided by the participants’ legal guardian/next of kin. The animal study was reviewed and approved by the Comité d’éthique en expérimentation animale Charles Darwin N°5, Institut du Cerveau et de la Moelle Epinière, Paris.

## Author Contributions

LPe, J-LM, ET, EI, and SD: study conception. J-LM, ET, RBe, EQ, and AH: study administration. IF, VG, TB, AB, HG, RBr, PG, FN, TP, MS, HW, RW, and JG: human study data acquisition. DP, AG, LPo, and JF: human study data QC. VG, EI, SF, CdM, and JG: mouse data acquisition. LPe, EI, EA, and ET: data analysis. LPe, EI, M-LP, ET, and J-LM: manuscript writing. M-LP, ET, EI, and J-LM: manuscript reviewing and editing. All authors contributed to the article and approved the submitted version.

## Conflict of Interest

The authors declare that the research was conducted in the absence of any commercial or financial relationships that could be construed as a potential conflict of interest.

## Publisher’s Note

All claims expressed in this article are solely those of the authors and do not necessarily represent those of their affiliated organizations, or those of the publisher, the editors and the reviewers. Any product that may be evaluated in this article, or claim that may be made by its manufacturer, is not guaranteed or endorsed by the publisher.

## Footnotes

1
http://adni.loni.usc.edu/methods/mri-analysis/mri-acquisition/

2
http://www0.cs.ucl.ac.uk/staff/g.ridgway/vbm/get_totals.m

3
https://genome.ucsc.edu

4
https://ldlink.nci.nih.gov/?tab=ldmatrix

5
http://www.dawba.info
