# Supplementary material for: Immune-Related Genetic Overlap Between Regional Gray Matter Reductions and Psychiatric Symptoms in Adolescents, and Gene-Set Validation in a Translational Model
Source: Front Syst Neurosci. 2021 Sep 30;15:725413. doi: 10.3389/fnsys.2021.725413 (PMC8514661; doi:10.3389/fnsys.2021.725413)
Supplement: Supplementary file 1 [file Data_Sheet_1.pdf]

Supplementary Materials

Supplementary Table S1: The 90 immune-related genes selected for this study, categorised according to their main function. For every gene, the number of related SNPs available in the IMAGEN database is given.

Gene	# SNPs	Gene	# SNPs
**Cytokines and Cytokine Receptors**		**Monocytosis and Granulopoiesis**	
IL1A	2	CXCL12	6
IL1B	3	TH	4
IL1R1	47	CXCR4	1
IL1R2	40	ADRB3	0
TNF	4	CSF1	9
LTA	5	CSF1R	30
TNFRSF1A	9	CSF2	2
TNFRSF1B	13	CSF2RA	0
IL6	5	CSF2RB	16
IL6R	9	CSF3	1
IFNG	3	CSF3R	8
IFNGR1	4	CCL2	3
IFNGR2	9	CCR2	0
IL12A	6	CCR4	2
IL12B	8		
IL12RB1	9	**Inflammatory Signaling Pathway**	
IL12RB2	10	NFKB1	13
IL2	1	NFKB2	3
IL2RA	35	RELA	3
IL2RB	16	RELB	3
IL2RG	1	REL	4
IL1RN	7	TRADD	0
IL4	2	RIPK1	5
IL4R	18	TRAF2	7
CXCL8	0	BIRC2	3
CXCR1	0	BIRC3	1
CXCR2	2	CHUK	2
IL10	7	IKBKB	4
IL10RA	6	IKBKG	1
IL10RB	10	MAPK8	5
IL11	3	MAPK9	16
IL13	3	MAPK14	15
IL13RA1	5	MAP2K7	3
IL13RA2	0	MAP3K2	4
IL18	5	MAP3K7	8
IL18R1	13	MAP3K8	6
IL18RAP	13	MAP4K2	2
IFNAR1	7		
IFNAR2	12	**Kynurenin Pathway**	
CRP	3	TDO	5
MIF	11	IDO1	2
IRF7	3	IDO2	20
IRF9	2	HAAO	11
		ACMSD	5
**Oxidative Stress Effectors**			
PTGS1	8	**Phospholipases**	
PTGS2	4	PLA2G4	27
NOS2	13	PLA2G6	13
MPO	2	PLA2G2A	4

Supplementary Table S2: The mean GM volumes (in ml) of different brain structures were obtained for the participants, both at BL (14 years old) and at FU2 (18 years old).

GM volume at BL (N = 1563)			GM volume at FU2 (N = 900)		
	**Mean (ml)**	**SD (ml)**		**Mean (ml)**	**SD (ml)**
**Hippocampus**	1.20	0.109	**Hippocampus**	1.18	0.109
**mPFC**	30.1	3.42	**mPFC**	28.4	3.27
**Amygdala**	1.86	0.186	**Amygdala**	1.87	0.192
**Striatum**	11.1	1.06	**Striatum**	10.8	1.03
**Cingulate**	20.6	2.30	**Cingulate**	19.0	2.12
**Visual cortex**	20.1	1.98	**Visual cortex**	18.6	1.94

Supplementary Table S3: The SNPs included in the HRRI. The minor and major allele as observed in this sample are described for every SNP as well as its minor allele frequency (MAF). For every SNP, the relative effect of one major allele on hippocampal GM volume (*B* (%)) and the corresponding p-value are given. Chr. = chromosome.

SNP	Gene	Chr.	Minor	Major	MAF	*B* (%)	*p*
**rs2243204**	IL13	5	T	C	0.0790	1.67	0.000228
**rs11465730**	IL18RAP	2	G	A	0.465	-0.774	0.00140
**rs132985**	PLA2G6	22	T	C	0.465	-0.751	0.00225
**rs10306163**	PTGS1	9	G	A	0.212	-0.923	0.00225
**rs917997**	IL18RAP	2	T	C	0.236	0.810	0.00476
**rs2546890**	IL12B	5	G	A	0.478	-0.637	0.00978
**rs10958713**	IKBKB	8	T	C	0.367	-0.652	0.0111
**rs3827354**	PLA2G6	22	T	C	0.394	0.624	0.0133
**rs3024685**	IL4R	16	C	T	0.386	0.607	0.0162
**rs12651699**	CSF1R	5	T	C	0.0945	-0.993	0.0169
**rs3761980**	MAPK14	6	G	A	0.0962	0.963	0.0197
**rs1003694**	IL2RB	22	T	C	0.277	0.639	0.0201
**rs3771150**	IL18RAP	2	A	G	0.286	-0.622	0.0201
**rs2284063**	PLA2G6	22	G	A	0.349	-0.593	0.0212
**rs2057768**	IL4R	16	T	C	0.300	0.617	0.0224
**rs4851527**	IL1R2	2	A	G	0.490	0.558	0.0236
**rs4936414**	IL10RA	11	C	T	0.340	-0.568	0.0279
**rs3755482**	IL1R2	2	G	A	0.285	-0.591	0.0294
**rs4851526**	IL1R2	2	G	A	0.409	-0.548	0.0310
**rs851019**	MAPK14	6	A	G	0.447	0.536	0.0313
**rs11256433**	IL2RA	10	G	T	0.221	-0.620	0.0334
**rs8178556**	IL10RB	21	G	T	0.0606	1.039	0.0377
**rs2495626**	IL13RA1	X	T	C	0.152	-0.583	0.0389
**rs2472394**	IKBKG	X	A	C	0.0835	0.751	0.0390
**rs2043055**	IL18	11	G	A	0.351	0.542	0.0412
**rs1433048**	IL12B	5	G	A	0.181	0.631	0.0463

Supplementary Table S4: The SNPs included in the MRRI. The minor and major allele as observed in this sample are described for every SNP as well as its minor allele frequency (MAF). For every SNP, the relative effect of one major allele on mPFC GM volume (*B* (%)) and the corresponding p-value are given. Chr. = chromosome.

SNP	Gene	Chr	Minor	Major	MAF	*B* (%)	*p*
**rs2287033**	IL18R1	2	C	T	0.479	-0.942	0.000462
**rs6770096**	CCR4	3	T	C	0.0863	1.489	0.00217
**rs12628766**	MIF	22	G	T	0.156	-1.160	0.00218
**rs2849997**	IL10RB	21	T	C	0.433	0.774	0.00447
**rs4848300**	IL1A	2	C	T	0.300	0.808	0.00531
**rs3213733**	IL18R1	2	A	C	0.180	-0.986	0.00636
**rs1558642**	IL1R1	2	T	G	0.226	0.875	0.00658
**rs11574625**	IL2RG	X	C	T	0.132	0.854	0.00763
**rs11570645**	PLA2G6	22	G	A	0.0572	-1.516	0.00816
**rs2247526**	IL10RB	21	C	T	0.443	0.691	0.00994
**rs3732131**	IL1R1	2	G	A	0.0736	1.292	0.0107
**rs4252287**	IL10RA	11	A	G	0.105	1.115	0.0109
**rs1860545**	TNFRSF1A	12	A	G	0.415	-0.682	0.0123
**rs7730091**	MAPK9	5	T	C	0.359	0.678	0.0132
**rs1035130**	IL18R1	2	T	C	0.286	0.687	0.0184
**rs12275349**	BIRC2	11	A	G	0.0886	1.023	0.0270
**rs3745383**	MAP2K7	19	T	C	0.136	-0.846	0.0279
**rs10306163**	PTGS1	9	G	A	0.212	-0.718	0.0292
**rs3095943**	MAPK9	5	T	C	0.0816	-1.032	0.0324
**rs949963**	IL1R1	2	A	G	0.184	-0.726	0.0395
**rs1534882**	CSF2RB	22	A	G	0.276	0.613	0.0419
**rs17027173**	IL18RAP	2	A	G	0.219	-0.640	0.0444
**rs20541**	IL13	5	A	G	0.189	-0.673	0.0494
**rs3917289**	IL1R1	2	T	G	0.076	-0.994	0.0528
**rs4601008**	MAPK9	5	G	A	0.146	0.692	0.0663
**rs10911933**	PLA2G4	1	C	T	0.179	-0.636	0.0670
**rs10905669**	IL2RA	10	T	C	0.227	0.564	0.0728
**rs2104286**	IL2RA	10	C	T	0.264	-0.518	0.0872
**rs132985**	PLA2G6	22	T	C	0.466	-0.447	0.0956

Supplementary Table S5: Candidate genes tested by RT-qPCR

Reference gene	Detector	Ct mean
Rab5a	*Csf1r*-Mm01266652_m1	26.90
Rab5a	*Ikbkb*-Mm01222247_m1	27.16
Rab5a	*Il1r2*-Mm00439629_m1	29.96
Rab5a	*Il2ra*-Mm01340213_m1	29.53
Rab5a	*Il2rb*-Mm00434268_m1	25.85
Rab5a	*Il4ra*-Mm01275139_m1	25.53
Rab5a	*Il10ra*-Mm00434151_m1	27.05
Rab5a	*Il10rb*-Mm00434157_m1	26.82
	Il12b-Mm01288989_m1	>36
	Il13-Mm00434204_m1	>37
Rab5a	*Il13ra1*-Mm01302068_m1	28.95
Rab5a	*Il18*-Mm00434226_m1	31.43
Rab5a	*Il18rap*-Mm00516053_m1	28.92
Rab5a	*Mapk14*-Mm01301009_m1	25.75
Rab5a	*Pla2g6*-Mm01299491_m1	29.48
Rab5a	*Ptgs1*-Mm00477214_m1	26.46
	Rab5a-Mm00727887_s1	28.05

Supplementary figure S1

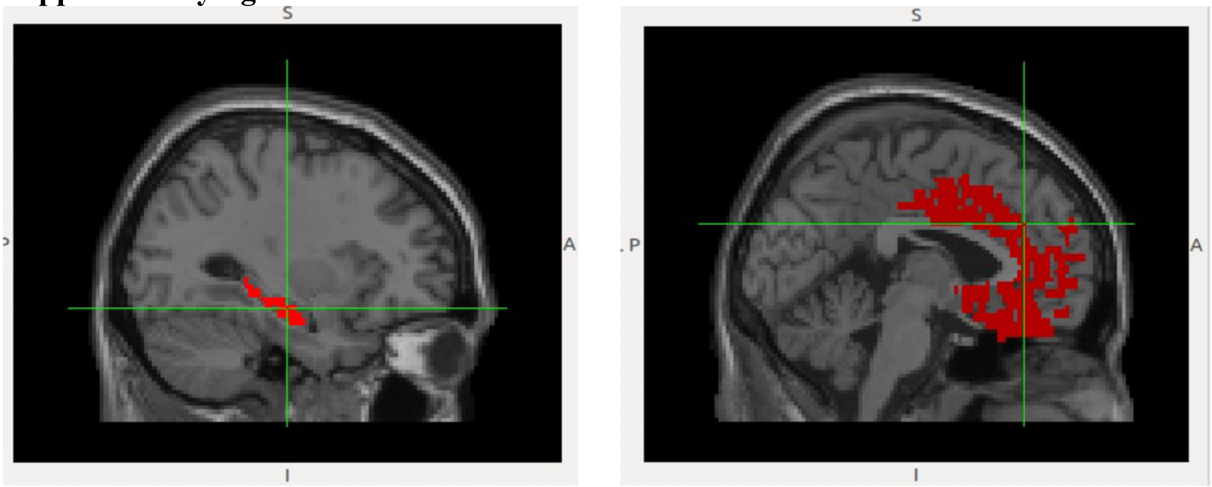

Regions of interest derived from masks of the hippocampus (left) and medial prefrontal cortex (right). Lateral rendering of the regional masks on the T1 MR image in one typical participant. The hippocampus was available from the WFU Pickatlas software, and the medial prefrontal cortex included the Brodmann areas 10, 11, 12, 14, 24, 25, 32 and 33.

Supplementary figure S2

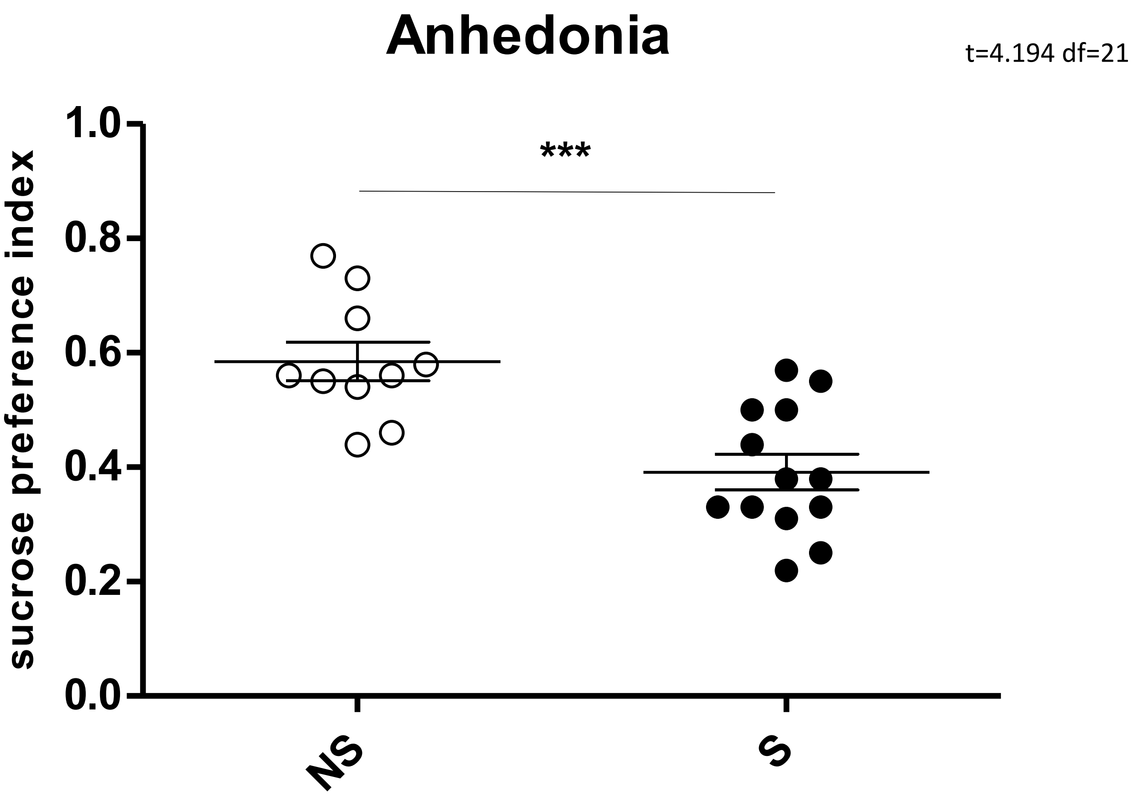

Early life stress diminishes sucrose preference in adolescent mice.
Newborn mice were either subjected to a maternal separation paradigm between P1 (post-natal day 1) and P14 (MS, black dots) or were left undisturbed (controls, NS, white dots). NS and MS mice were evaluated for anhedonia (measured in the sucrose preference test) in late adolescence, P52. Two-tailed Student's t-test shows increased levels of anhedonia (df=21; t=4.194) evidenced as reduced sucrose preference in MS mice as compared to NS. ***p<0.0001.

Supplementary figure S3

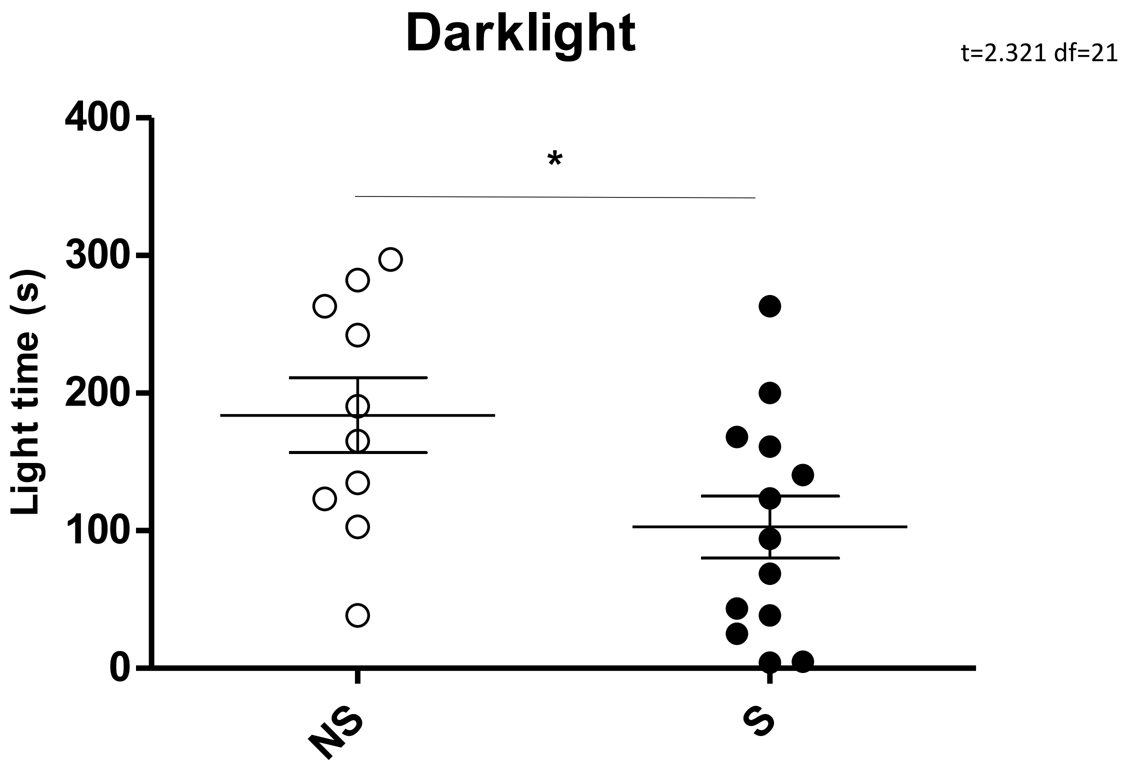

Early life stress increases time spent in the dark in adolescent mice.
Newborn mice were either subjected to a maternal separation paradigm between P1 (post-natal day 1) and P14 (MS, black dots) or were left undisturbed (controls, NS, white dots). NS and MS mice were evaluated for anxiety (measured in the dark-light test) in late adolescence, P59. Two-tailed Student's t-test shows increased levels of anxiety (df=21; t=2.321) evidenced as increased tme spent in the dark in MS mice as compared to NS. * $p<0.05$

Supplementary Figure S4

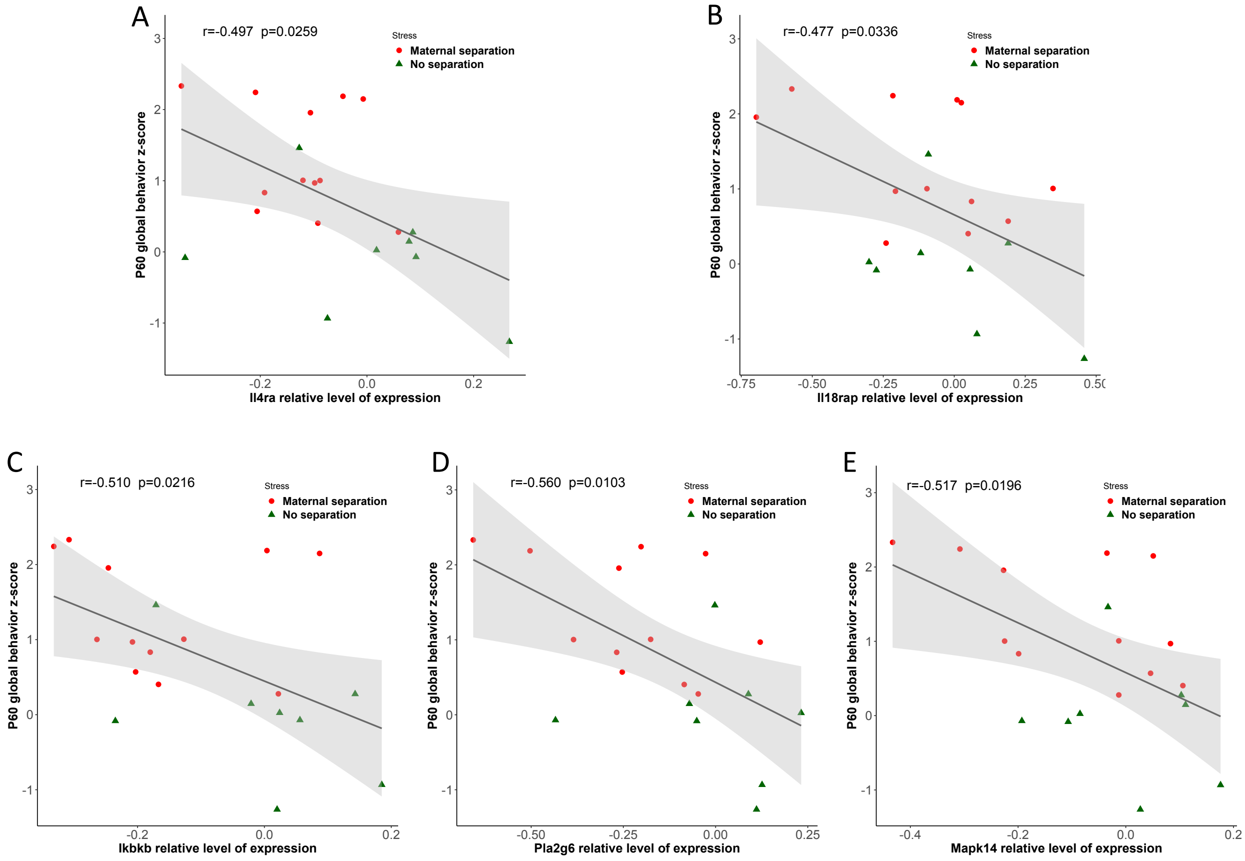

Link between HRRI candidate gene levels of transcriptional expression and depressive-like behavior.

Linear regressions with 95% confidence intervals (in gray) are plotted between the depression-index and the *Il4ra* (A), *Il18rap* (B), *Ikbkb* (C), *Pla2g6* (D), *Mapk14* (E) transcriptional relative levels obtained by RT-qPCR from blood in MS (red circles) and NS (green triangles) adolescent mice. The Pearson correlation coefficient and the associated *p-value* are indicated.

.
